# Fulham Infirmary

**Published:** 1913-01-04

**Authors:** 


### FULHAM INFIRMARY.

The annual performance by the staff of the Fulham Infirmary will take place on the evenings of January 9 and January 10, beginning at 7.30 P.M. The medical and nursing staff hope that any former medical officers or nurses who can attend on either evening will do so.

